# Evaluating Completeness of Admissions Clerking Procedure for Patients Admitted to an Acute Psychiatric Inpatient Unit

**DOI:** 10.1192/bjo.2022.311

**Published:** 2022-06-20

**Authors:** Neha Karthikeyan, Charlotte Little, Anjali Nair, Andrew Sandor

**Affiliations:** Sussex Partnership NHS Foundation Trust, Chichester, United Kingdom

## Abstract

**Aims:**

There is a well-known bidirectional relationship between mental and physical health. A thorough mental and physical health assessment of new inpatients on admission is crucial to assess possible organic causes of psychiatric presentations and enable appropriate treatment to be commenced in a timely manner. We noted a pattern of incomplete clerking, leading to delays in commencing treatment and increased workload pressure on the ward medical team. Our audit aims to assess how thoroughly and in what time frame patients' admission assessments are being completed on an acute psychiatric inpatient unit.

**Methods:**

A sample of 20 patients was used for each cycle. Eight components were identified from the Trust clerking procedure. These were a full psychiatric history, capacity assessment, prescription chart, physical health systems review, physical examination, blood tests, ECG and VTE assessment. The expected standard was completion of all of the above at the point of admission or, if applicable, adequate documentation of patient refusal with documented reattempt within 24 hours.

Electronic patient records were retrospectively reviewed to identify whether each component was completed, the number of days from admission to completion and if completed by the clerking doctor. Evidence of documented patient refusals and the number of incomplete draft entries were noted.

**Results:**

Initial results showed only 20% of patients (n = 4) had the full expected clerking procedure completed at admission. Physical health assessments (ECG, blood test, physical examination) had the lowest completion rates. When components were not completed by the clerking doctor, there was often a delay of several days from admission to completion of by the ward day team. For 50% of patients, some assessments were never completed (without adequate refusal documented). Eight patients did not have a completed VTE assessment by the point of discharge.

**Conclusion:**

Reasons were identified for the poor completion rates of clerking assessments. These included a lack of understanding by on-call doctors about the importance of completing all assessments, unfamiliarity with ward equipment, lack of adequate handover to the ward medical team and staffing pressures. A new handover checklist was introduced for clerking doctors to use and enable better communication with the ward team. Clerking procedure summaries were displayed in on-call rooms and additional teaching was organised.

